# Optimization of the Trajectory, Transmit Power, and Power Splitting Ratio for Maximizing the Available Energy of a UAV-Aided SWIPT System

**DOI:** 10.3390/s22239081

**Published:** 2022-11-23

**Authors:** Gitae Park, Kisong Lee

**Affiliations:** Department of Information and Communication Engineering, Dongguk University, Seoul 04620, Republic of Korea

**Keywords:** unmanned aerial vehicle, energy harvesting, trajectory, SWIPT, convex optimization

## Abstract

In this study, we investigate the maximization of the available energy for an unmanned aerial vehicle (UAV)-aided simultaneous wireless information and power transfer (SWIPT) system, in which the ground terminals (GTs) decode information and collect energy simultaneously from the downlink signal sent by the UAV based on a power splitting (PS) policy. To guarantee that each GT has a fair amount of available energy, our aim is to optimize the trajectory and transmit power of the UAV and the PS ratio of the GTs to maximize the minimum average available energy among all GTs while ensuring the average spectral efficiency requirement. To address the nonconvexity of the formulated optimization problem, we apply a successive convex optimization technique and propose an iterative algorithm to derive the optimal strategies of the UAV and GTs. Through performance evaluations, we show that the proposed scheme outperforms the existing baseline schemes in terms of the max–min available energy by adaptively controlling the optimization variables according to the situation.

## 1. Introduction

Unmanned aerial vehicles (UAVs) have attracted considerable attention as a promising technology for providing a high quality-of-service for a variety of applications, such as surveillance, emergency, and parcel deliveries [1]. Due to their high mobility and flexibility, UAVs are increasingly being used in wireless communications. Moreover, UAVs typically form a line-of-sight (LoS) air-to-ground wireless link, providing a high data rate for communication applications [2].

Accordingly, early studies have examined the optimization of UAV placement, including the altitude and horizontal location [3,4]. In addition, considering the high mobility of UAVs, the design of UAV trajectories that can adapt to situations has also been investigated [5,6]. Recently, UAV-enabled wireless power transfer (WPT) has been researched for maximizing the amount of energy transferred from the UAV to ground terminals (GTs). This is possible because UAVs can transmit energy with high efficiency by forming an LoS link [7]. To transfer information and energy at the same time, the studies have been extended to examine the UAV-aided wireless powered communication network (WPCN) and simultaneous wireless information and power transfer (SWIPT) systems. In particular, for UAV-aided WPCN, where the UAV broadcasts wireless energy to charge GTs and the GTs send data to the UAV using the harvested energy, a weighted harvest-then-transmit protocol has been proposed to maximize the sum throughput [8]. Furthermore, a UAV hovering strategy has been devised to maximize the minimum average rate of the GTs [9]. Given that many components required to harvest energy from radio frequency (RF) signals can be shared with the components of wireless communications, e.g., antenna, diode, and low-pass filter, the concept of SWIPT has been proposed [10] that allows wireless devices to recharge batteries from the RF signals while information decoding by dividing the received signals. Accordingly, for UAV-aided SWIPT, where GTs receive information and energy simultaneously from the downlink signal of the UAV, an emergency communications framework has been established that considers trajectory planning and resource scheduling [11]. A joint optimization of the trajectory, transmit power, and power splitting ratio was also investigated to maximize the minimum average rate [12]. Furthermore, the problem of harvested energy maximization was considered for the Internet-of-Things (IoT) [13] and multiuser relaying systems [14]. Some recent works also considered a satellite and aerial-integrated network with beamforming design [15,16] and rate-splitting multiple access [17]. Moreover, the studies on intelligent reflecting surface empowered UAV-aided SWIPT systems were investigated under perfect channel state information (CSI) [18] and statistical CSI [19]. The energy efficiency optimization problem was also considered for the device-to-device communications underlaying UAV-assisted IoT networks with SWIPT [20]. Although various investigations have been undertaken on UAV-aided SWIPT systems [11,12,13,14], no studies have been conducted on providing fair energy to GTs by considering their different residual energies.

To support GTs in receiving the available energy fairly, we investigate an effective UAV trajectory and resource allocation strategy for UAV-aided SWIPT systems based on a power splitting (PS) policy. Compared to previous studies on the rate maximization [12] or the energy harvesting (EH) maximization [13,14] of UAV-aided SWIPT systems, the main contributions of our work can be summarized as follows:

- For reliable subsequent processing of GTs, it is important to ensure an equitable amount of available energy for GTs with different residual energies. Therefore, we formulate the problem to maximize the minimum average available energy among all GTs while guaranteeing the average spectral efficiency (SE) requirement.
- To solve the nonconvex optimization problem, we transform the original problem into a tractable convex form using a successive convex optimization technique. Based on the transformed problem, the optimal trajectory and transmit power of the UAV and the PS ratio of the GTs are effectively found by the proposed iterative algorithm.
- Through performance evaluations, we show that the proposed UAV trajectory for ensuring fair available energy is completely different from the trajectory of the existing algorithms. We also verify that the proposed scheme supports the highest max–min available energy for GTs compared to state-of-the-art baseline schemes by adaptively controlling the optimization variables.

The remainder of this paper is organized as follows: In Section 2, we present the model of the UAV-aided SWIPT system, complete with the problem statement. In Section 3, we solve a nonconvex problem and propose an iterative algorithm for optimizing the trajectory, transmit power, and PS ratio. In Section 4, we report on the performance evaluation, and the conclusions are presented in Section 5.

## 2. System Model and Problem Statement

**Figure 1:**
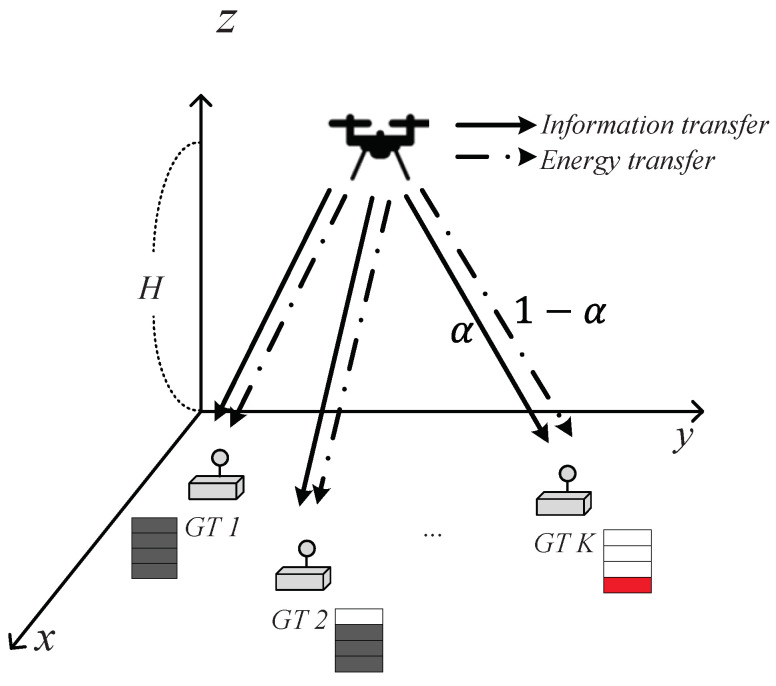
System model of a UAV-aided downlink SWIPT.

As shown in Figure 1, we considered a UAV-aided downlink SWIPT system, in which the UAV broadcasts data signals to *K* GTs, and each GT receives information and harvests energy simultaneously from this signal using a PS policy. We denote K={1,2,⋯,K} as the set of GTs with |K|=K, and each GT has a fixed location on the ground, such as $w_{k}={\left[x_{k},y_{k}\right]}^{T}\in R^{2\times 1},\;k\in K$. To ensure reliable SWIPT functionality for GTs, the UAV flies with a fixed altitude *H* and finite flight time *T*, which is equally divided into *N* time slots, $\delta =\frac{T}{N}$, and N={1,2,⋯,N}. Here, *N* is assumed to be sufficiently large that the position of the UAV can be considered static within each time slot. The horizontal location of the UAV at each time slot is denoted by $q\left[n\right]={\left[x\left[n\right],y\left[n\right]\right]}^{T}\in R^{2\times 1},\;n\in N$. Given that the maximum flying speed of the UAV is *V*, the maximum flying distance in each time slot is limited to L=Vδ. In addition, the UAV must return to its starting position periodically after one period. Therefore, the constraints of UAV mobility are represented by

(1) $$\begin{array}{cc} ∥q[n+1]-q[n]∥ & \leq L,\;\;\forall n\in N\setminus \{N\}, \end{array}$$

(2) $$\begin{array}{cc} q[1] & =q[N]. \end{array}$$

The UAV also has the following average and peak power constraints:

(3) $$\begin{array}{cc} \frac{1}{N}\sum_{n\in N}p\left[n\right] & \leq P_{\mathrm{avg}}, \end{array}$$

(4) $$\begin{array}{cc} 0\leq p[n] & \leq P_{\mathrm{peak}},\;\;\forall n, \end{array}$$

where p[n] indicates the transmit power of the UAV in time slot *n*, and $P_{\mathrm{avg}}$ and $P_{\mathrm{peak}}$ are the average and peak power budgets for the UAV, respectively.

For simplicity, the free-space path loss model is adopted [11,12,13,14], in which the air-to-ground channels are assumed to be dominated by LoS links, and the Doppler effect due to the UAV mobility is assumed to be perfectly compensated at the GTs. Then, the channel gain from the UAV to GT *k* in time slot *n* is given by

(5) $$\begin{array}{c} h_{k}\left[n\right]=\frac{\beta_{0}}{d_{k}^{2}\left[n\right]}=\frac{\beta_{0}}{∥q\left[n\right]-w_{k}{∥}^{2}+H^{2}},\;\;\forall k,\;n, \end{array}$$

where $\beta_{0}$ represents the channel power gain at the unit reference distance, and $d_{k}\left[n\right]$ is the distance between the UAV and GT *k* in time slot *n*.

Each GT splits the received signal to receive information in parts $\alpha_{k}\left[n\right]$ and harvest energy in the remaining parts $1-\alpha_{k}\left[n\right]$ in each time slot. Therefore, the PS ratio constraint is expressed as

(6) $$\begin{array}{c} 0\leq \alpha_{k}\left[n\right]\leq 1,\;\;\forall k,\;n. \end{array}$$

Then, the achievable SE from the UAV to GT *k* in time slot *n* is represented by

(7) $$\begin{array}{cc} R_{k}\left[n\right] & =\log_{2}\left(1+\frac{\alpha_{k}\left[n\right]h_{k}\left[n\right]p\left[n\right]}{\alpha_{k}\left[n\right]\sigma_{A}^{2}+\sigma^{2}}\right)\\ \; & =\log_{2}\left(1+\frac{\beta_{0}\alpha_{k}\left[n\right]p\left[n\right]}{\left(\alpha_{k}\left[n\right]\sigma_{A}^{2}+\sigma^{2}\right)(∥q\left[n\right]-w_{k}{∥}^{2}+H^{2})}\right), \end{array}$$

where $\sigma^{2}$ and $\sigma_{A}^{2}$ are the baseband noise power and antenna noise power, respectively. The average SE from the UAV to GT *k* over the whole period is expressed as

(8) $$\begin{array}{cc} {\bar{R}}_{k} & =\frac{1}{N}\sum_{n\in N}R_{k}\left[n\right]. \end{array}$$

For reliable communication between the UAV and GT *k*, we consider the following average SE requirement for each GT:

(9) $$\begin{array}{c} {\bar{R}}_{k}\geq R_{\min},\;\;\forall k, \end{array}$$

where $R_{\min}$ is the minimum required SE.

The harvested energy of GT *k* in time slot *n* is obtained as

(10) $$\begin{array}{cc} E_{k}\left[n\right] & =\delta \eta_{k}\left(1-\alpha_{k}\left[n\right]\right)h_{k}\left[n\right]p\left[n\right]\\ \; & =\frac{\gamma_{k}\left(1-\alpha_{k}\left[n\right]\right)p\left[n\right]}{∥q\left[n\right]-w_{k}{∥}^{2}+H^{2}}, \end{array}$$

where $\eta_{k}$ is the energy conversion efficiency of GT *k*, and $\gamma_{k}$ is defined as $\gamma_{k}=\delta \beta_{0}\eta_{k}$. Then, the average available energy of GT *k* including its residual energy $E_{k}^{r}$ and average harvested energy over *T* is given by

(11) $$\begin{array}{cc} {\bar{E}}_{k} & =E_{k}^{r}+\frac{1}{N}\sum_{n\in N}E_{k}\left[n\right]. \end{array}$$

To support each GT receiving a fair amount of available energy, we aim to optimize the trajectory Q≜{q[n],∀n} and the transmit power P≜{p[n],∀n} of the UAV jointly with the PS ratio of the GTs $A≜\{\alpha_{k}\left[n\right],\;\forall k,\;n\}$ to maximize the minimum average available energy among all GTs while ensuring the average SE requirement for each GT, which can be formulated as follows:

(12) $$\begin{array}{cc} (P0):\;\max_{Q,\;P,\;A} & \;\;\;\;\min_{k\in K}{\bar{E}}_{k}\\ \mathrm{such}\mathrm{that} & \;\;\;\;\;(1)–(6),\;\mathrm{and}\;(9). \end{array}$$

## 3. Proposed Algorithm

In (12), the objective function and the constraint (9) are not jointly concave with respect to Q, P, and A; hence, the optimization problem (P0) is nonconvex. Therefore, we divide the original problem into subproblems that are convex with respect to each optimization variable through relaxation and solve it by fixing the remaining variables.

### 3.1. Transmit Power Optimization

For a fixed A and Q, by introducing an auxiliary variable $E_{\min}$ to represent the lower bound of the original objective function in the problem (P0), the original problem can be reformulated for a single variable P as follows:

(13) $$\begin{array}{cc} (P1):\;\; & \max_{P,\;E_{\min}}\;\;\;\;E_{\min}\\ \; & \;\;s.t.\;\;\;\;\;{\bar{E}}_{k}\geq E_{\min},\;\;\forall k\\ \; & \;\;\;\;\;\;\;\;\;\;\;\;\;(3),\;(4),\;\mathrm{and}\;(9). \end{array}$$

Given that problem (P1) is concave with respect to P, it can be easily solved by existing convex solvers, such as the interior point method.

### 3.2. Power Splitting Ratio Optimization

For a determined P, we adopt a successive convex optimization technique to find the PS ratio efficiently, where the original function can be approximated by a tractable function at a given point in each iteration. Initially, $R_{k}\left[n\right]$ in (7) can be rewritten as

(14) $$\begin{array}{cc} R_{k}\left[n\right] & =\log_{2}\left(\alpha_{k}\left[n\right]\left(h_{k}\left[n\right]p\left[n\right]+\sigma_{A}^{2}\right)+\sigma^{2}\right)-{\hat{R}}_{k}\left[n\right], \end{array}$$

where ${\hat{R}}_{k}\left[n\right]$ is given by

(15) $$\begin{array}{cc} {\hat{R}}_{k}\left[n\right] & =\log_{2}\left(\alpha_{k}\left[n\right]\sigma_{A}^{2}+\sigma^{2}\right)\\ \; & \overset{\left(a\right)}{\leq }\log_{2}\left(\alpha_{k}^{\left(m\right)}\left[n\right]\sigma_{A}^{2}+\sigma^{2}\right)+\frac{\sigma_{A}^{2}}{\ln2\left(\alpha_{k}^{\left(m\right)}\left[n\right]\sigma_{A}^{2}+\sigma^{2}\right)}\left(\alpha_{k}\left[n\right]-\alpha_{k}^{\left(m\right)}\left[n\right]\right)\\ \; & ≜{\hat{R}}_{k}^{\mathrm{UB}}\left[n\right], \end{array}$$

where $\alpha_{k}^{\left(m\right)}\left[n\right]$ is the PS ratio of GT *k* in the time slot *n* for *m*-th iteration. The inequality (a) in (15) is obtained by the first-order Taylor expansion, because a concave function is upper bounded by its first-order Taylor expansion at any point.

Using the upper bound of ${\hat{R}}_{k}\left[n\right]$, which is denoted as ${\hat{R}}_{k}^{\mathrm{UB}}\left[n\right]$, the concave lower bound of $R_{k}\left[n\right]$ can be derived as follows:

(16) $$\begin{array}{cc} R_{k}^{\mathrm{LB}}\left[n\right] & =\log_{2}\;\left(\alpha_{k}\left[n\right]\left(h_{k}\left[n\right]p\left[n\right]+\sigma_{A}^{2}\right)+\sigma^{2}\right)-{\hat{R}}_{k}^{\mathrm{UB}}\left[n\right]. \end{array}$$

Then, for a fixed P and Q, the original problem can be reformulated for a single variable A as follows:

(17) $$\begin{array}{cc} (P2):\;\; & \max_{A,\;E_{\min}}\;\;\;\;E_{\min}\\ \; & \;\;s.t.\;\;\;\;\;{\bar{E}}_{k}\geq E_{\min},\;\;\forall k\\ \; & \;\;\;\;\;\;\;\;\;\;\;\;\;\frac{1}{N}\sum_{n\in N}R_{k}^{\mathrm{LB}}\left[n\right]\geq R_{\min},\;\;\forall k\\ \; & \;\;\;\;\;\;\;\;\;\;\;\;\;(6). \end{array}$$

The problem (P2) is concave with respect to A; it can also be solved effectively by convex solvers.

### 3.3. Trajectory Optimization

For a determined P and A, the original problem is still not concave with respect to Q due to the nonconvexity of the objective function and the constraint (9). To address the nonconvexity of the objective function, we also find the lower bound of $E_{k}\left[n\right]$ using the first-order Taylor expansion, as follows:

(18) $$\begin{array}{cc} E_{k}\left[n\right] & \geq -\frac{\gamma_{k}\left(1-\alpha_{k}\left[n\right]\right)p\left[n\right]}{{\left(∥q^{\left(m\right)}\left[n\right]-w_{k}{∥}^{2}+H^{2}\right)}^{2}}\left(∥q\left[n\right]-w_{k}{∥}^{2}-{∥q^{\left(m\right)}\left[n\right]-w_{k}∥}^{2}\;\right)+\frac{\gamma_{k}\left(1-\alpha_{k}\left[n\right]\right)p\left[n\right]}{∥q^{\left(m\right)}\left[n\right]-w_{k}{∥}^{2}+H^{2}}\\ & ≜E_{k}^{\mathrm{LB}}\left[n\right], \end{array}$$

where $q^{\left(m\right)}\left[n\right]$ is the trajectory of UAV in time slot *n* for the *m*-th iteration.

Similarly, to address the nonconvexity of constraint (9), the lower bound of $R_{k}\left[n\right]$ can be derived by the first-order Taylor expansion as follows:

(19) $$\begin{array}{cc} R_{k}\left[n\right] & \geq -A_{k}^{\left(m\right)}\left[n\right]\;\left(∥q\left[n\right]\;-\;w_{k}{∥}^{2}\;-\;{∥q^{\left(m\right)}\left[n\right]\;-\;w_{k}∥}^{2}\right)\;+\;\log_{2}\left(\;1\;+\;\frac{\beta_{0}\alpha_{k}\left[n\right]p\left[n\right]}{\left(\alpha_{k}\left[n\right]\sigma_{A}^{2}\;+\;\sigma^{2}\right)(∥q^{\left(m\right)}\left[n\right]\;-\;w_{k}{∥}^{2}\;+\;H^{2})}\;\right)\\ \; & ≜R_{k}^{\mathrm{LB}}\left[n\right], \end{array}$$

where $A_{k}^{\left(m\right)}\left[n\right]$ is defined as

(20) $$\begin{array}{cc} A_{k}^{\left(m\right)}\left[n\right] & =\frac{\frac{\beta_{0}\alpha_{k}\left[n\right]p\left[n\right]}{{\left(∥q^{\left(m\right)}\left[n\right]-w_{k}{∥}^{2}+H^{2}\right)}^{2}}}{\ln2\left(\frac{\beta_{0}\alpha_{k}\left[n\right]p\left[n\right]}{∥q^{\left(m\right)}\left[n\right]-w_{k}{∥}^{2}+H^{2}}+\alpha_{k}\left[n\right]\sigma_{A}^{2}+\sigma^{2}\right)}. \end{array}$$

Therefore, for a fixed P and A, the original problem can be reformulated for a single variable Q, as follows:

(21) $$\begin{array}{cc} (P3):\;\; & \max_{Q,\;E_{\min}}\;\;\;\;E_{\min}\\ \; & \;\;s.t.\;\;\;\;\;E_{k}^{r}+\frac{1}{N}\sum_{n\in N}E_{k}^{\mathrm{LB}}\left[n\right]\geq E_{\min},\;\;\forall k\\ \; & \;\;\;\;\;\;\;\;\;\;\;\;\;\frac{1}{N}\sum_{n\in N}R_{k}^{\mathrm{LB}}\left[n\right]\geq R_{\min},\;\;\forall k\\ \; & \;\;\;\;\;\;\;\;\;\;\;\;\;(1)\;\mathrm{and}\;(2). \end{array}$$

With the concave lower bounds, $E_{k}^{\mathrm{LB}}\left[n\right]$ and $R_{k}^{\mathrm{LB}}\left[n\right]$, the problem (P3) is concave with respect to Q. Therefore, the problem can be solved using standard convex optimization solvers, such as CVX [21].

To solve the nonconvex problem (P0), we develop three subproblems that are concave with respect to each optimization variable, and then iteratively solve each subproblem using a convex solver until convergence to find the optimal variables. Algorithm 1 lists the detailed procedure for the proposed algorithm. In addition, the convergence of Algorithm 1 can be guaranteed because the objective function of each subproblem is non-decreasing after each update, meaning that it is bounded by a finite value. It is known that the number of iterations required for the convergence of the interior point method for the worst-case is $O\;\left(\sqrt{s}\log\left(1/\varepsilon \right)\right)$, where *s* is the number of variables to be optimized and ε>0 is the threshold for convergence. Moreover, the number of calculations in each iteration is $O\;\left(s^{3}\right)$ [22,23]. Based on this result, the computational complexity of Algorithm 1 can be derived as $O\;(M{\left(KN\right)}^{3.5}\log\left(1/\varepsilon \right))$, where *M* is the number of iterations for the outer loop (lines 2–7), which implies that the proposed algorithm has a polynomial complexity of *K* and *N*.
**Algorithm 1** Proposed Algorithm1: Initialize $P^{\left(m\right)}$, $A^{\left(m\right)}$, $Q^{\left(m\right)}$, and m=02: **repeat**3:  Find $P^{\left(m+1\right)}$ by solving (P1) for given $\left\{P^{\left(m\right)},A^{\left(m\right)},Q^{\left(m\right)}\right\}$4:  Find $A^{\left(m+1\right)}$ by solving (P2) for given $\left\{P^{\left(m+1\right)},A^{\left(m\right)},Q^{\left(m\right)}\right\}$5:  Find $Q^{\left(m+1\right)}$ by solving (P3) for given $\left\{P^{\left(m+1\right)},A^{\left(m+1\right)},Q^{\left(m\right)}\right\}$6:  Update m←m+17: **until** Convergence

## 4. Performance Evaluations and Discussion

To evaluate the performance of the proposed scheme, we considered the following system parameters as the default values, unless otherwise stated [11,12,13,14]: T=80 s, δ=0.5 s, K=5, H=50 m, V=25 m/s, $P_{\mathrm{avg}}=40$ dBm, $P_{\mathrm{peak}}=4P_{\mathrm{avg}}$, $R_{\min}=8$ bps/Hz, $\eta_{k}=0.5,\;\forall k$, $\beta_{0}=0$ dB, $\sigma_{A}^{2}=-70$ dBm, and $\sigma^{2}=-40$ dBm. We also distributed GTs that had different residual energies, e.g., $\left[E_{1}^{r},E_{2}^{r},E_{3}^{r},E_{4}^{r},E_{5}^{r}\right]=\left[0.3,0.2,0.005,0,0.1\right]$ mW, over an area of 400×400 m and compared the performance of the following schemes:

- Proposed scheme: The trajectory and transmit power of the UAV and the PS ratio of the GTs were obtained using Algorithm 1.
- Max–min rate scheme: The trajectory and transmit power of the UAV and the PS ratio of the GTs were found to maximize the minimum average rate among all GTs, ensuring the average harvested energy requirement [12].
- Fixed EH scheme: The trajectory and transmit power of the UAV were found to maximize the minimum available energy among all GTs, ensuring the average SE requirement. However, the PS ratio of the GTs was fixed as $\alpha_{k}\left[n\right]=0.8$ [13].
- Hover-and-fly scheme: The UAV hovered over the GTs’ positions sequentially and flew in a straight line from each user to the other at a constant speed. The transmit power of the UAV and the PS ratio of the GTs were determined in the same way as the proposed method.
- Circular scheme: The UAV had a circular trajectory with a radius of 100 m centered on the geometric mean of the GTs’ positions. The transmit power of the UAV and the PS ratio of the GTs were determined in the same way as the proposed method.

**Figure 2:**
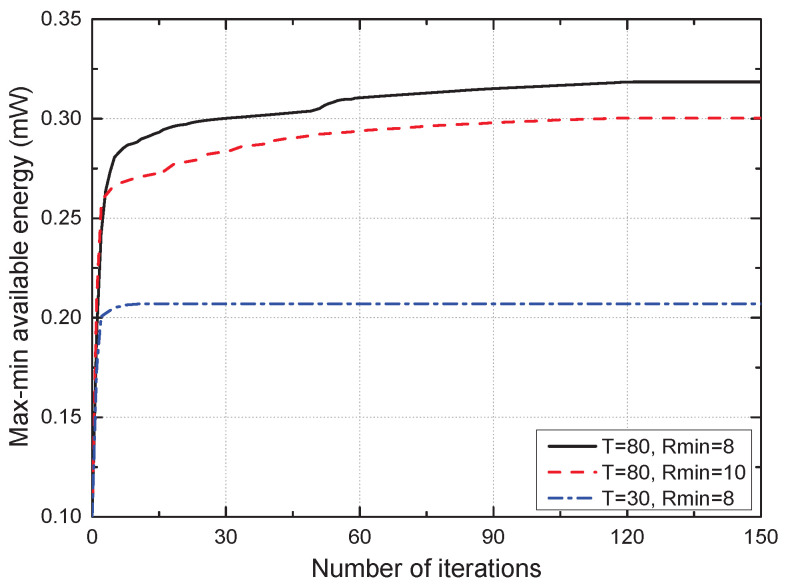
Convergence of the proposed scheme.

Figure 2 shows the convergence performance of the proposed scheme for different values of *T* and $R_{\min}$. For each Q, P, and A, the number of variables to optimize was $N=\frac{T}{\delta }$, which increased with *T*. Therefore, when *T* was small, e.g., T=30 s, the proposed algorithm converged to a stationary point faster because the number of optimization variables was the smallest. Moreover, when *T* was sufficiently large, e.g., T=80 s, the smaller the $R_{\min}$, the greater the convergence point of the proposed algorithm. This is because the GT used less energy to ensure the average SE requirement when $R_{\min}$ was small; hence, it could collect a large amount of available energy. This result confirmed the stable convergence of the proposed algorithm for different values of *T* and $R_{\min}$.

**Figure 3:**
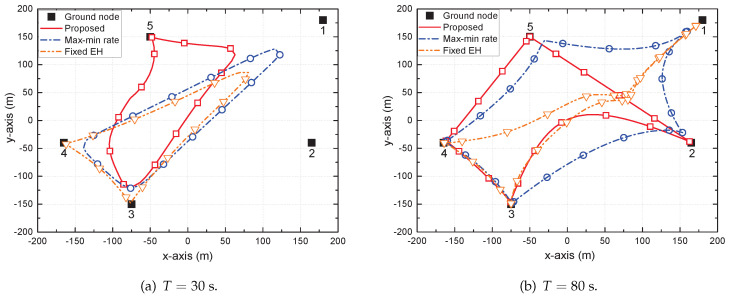
Trajectory of the UAV for different values of *T*. Upload modified figure files as R22 and R33 in Fig2 folder.

Figure 3 shows the trajectory of the UAV for different values of *T*, e.g., T=30 s and T=80 s. The trajectories of the hover-and-fly and circular schemes were omitted because their trajectories were fixed. By comparing with the trajectory of the max–min rate scheme, the difference between the trajectories for maximizing EH and maximizing the rate was confirmed. Moreover, by comparing with the trajectory of the fixed EH scheme, the effect of optimizing the PS ratio was verified. The circular, square, and triangular markers indicate the positions of the UAV sampled every 2.5 s. When T=30 s, the UAV flew close to its maximum speed *V* to support shorter LoS links for each GT, although it could not visit all the GTs directly due to the limited time period. As *T* increased, e.g., T=80 s, the UAV moved closer to the GTs by extending its trajectory to support them efficiently. In particular, for the max–min rate scheme, the UAV visited all GTs to equally support the average SE, provided the average harvested energy requirement was satisfied. For the fixed EH scheme, the UAV directly visited GTs 3 and 4, which had the least available energy, but not GTs 1, 2, and 5. Instead, it stayed in the middle of GTs 1, 2, and 5 for a long period to provide a fair amount of available energy. This is because GTs cannot adaptively adjust their PS ratio depending on the situation. However, for the proposed scheme, the UAV directly visited all GTs except GT 1, which already had enough available energy, and the GTs harvested sufficient energy by controlling the PS ratio when the UAV was close to them.

**Figure 4:**
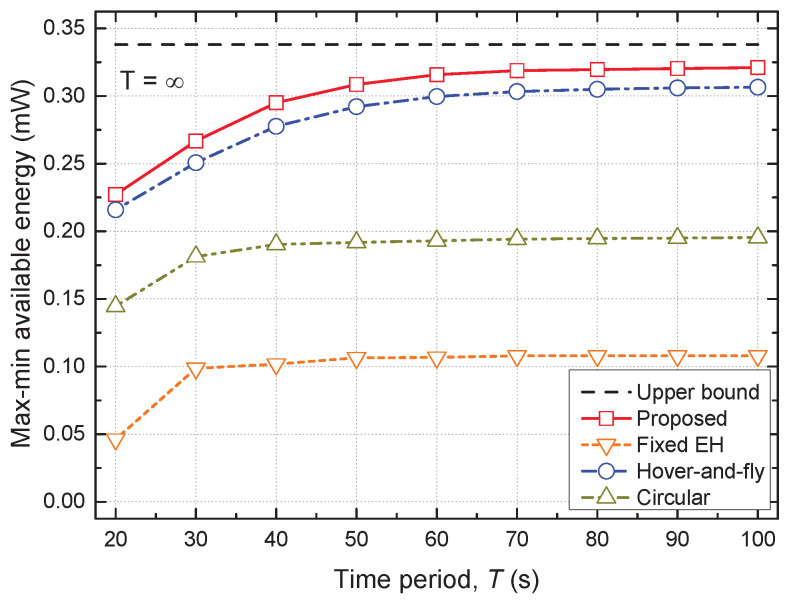
Max–min available energy vs. time period.

Figure 4 shows the max–min available energy versus time period *T* for all the considered schemes. The max–min available energy of all the schemes increased with *T* and eventually saturated when *T* was sufficiently large. Specifically, the max–min available energy of the proposed scheme approached the upper bound of T=∞ as *T* increased. This was because the UAV could fly for a longer period and had a chance to be closer to the GTs. The circular and fixed EH schemes exhibited significantly lower performance than the proposed scheme. This was because the circular scheme could not directly visit the GTs due to its predetermined trajectory and the fixed EH scheme could not control the PS ratio adaptively. The hover-and-fly scheme, where all GTs were visited sequentially, achieved the highest performance among the conventional baseline schemes, which demonstrated the importance of reaching the GTs directly for reliable EH. Moreover, the proposed scheme outperformed the conventional schemes, and this performance gap increased with *T*.

**Figure 5:**
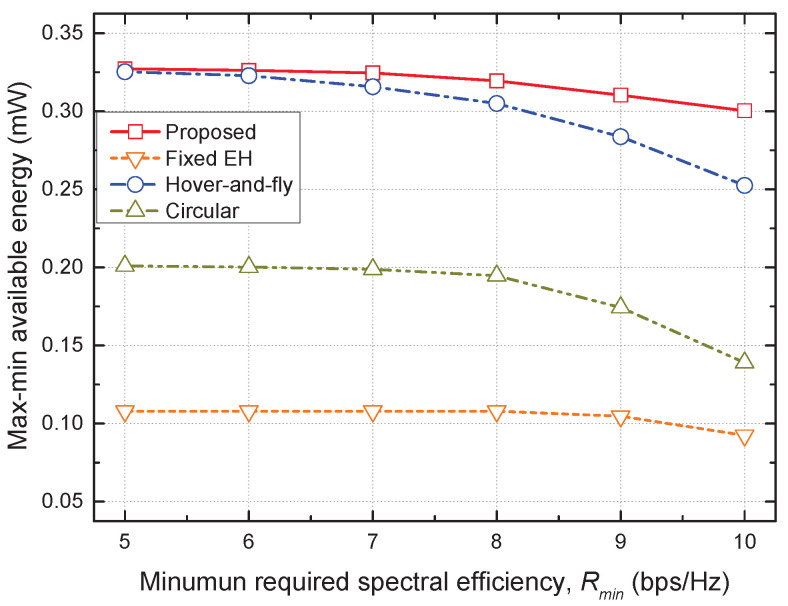
Max–min available energy vs. minimum required spectral efficiency.

Figure 5 shows the max–min available energy versus the minimum required SE $R_{\min}$ for all the considered schemes. As the $R_{\min}$ increased, each GT needed to use a large proportion of the received signal to decode information to guarantee the minimum SE requirement, reducing the available energy for all schemes. In addition, for a larger $R_{\min}$, the hover-and-fly scheme could not support a fair amount of available energy for GTs that had different residual energies. This was because the UAV always visited all the GTs, rather than optimizing its trajectory according to the situation. As a result, the performance gap between the proposed and hover-and-fly schemes increased as $R_{\min}$ increased. Finally, the proposed scheme achieved the highest max–min available energy over the complete range of $R_{\min}$, verifying the superiority of the proposed trajectory and resource allocation scheme.

## 5. Conclusions

For the UAV-aided downlink SWIPT system with a PS policy, we jointly optimized the trajectory and transmit power of the UAV and the PS ratio of the GTs to maximize the minimum average available energy among all GTs, while ensuring the constraint of the average SE for each GT. We converted the original nonconvex problem into a tractable convex form using the successive convex optimization technique and proposed an iterative algorithm to find the optimal resource allocation strategy. Through performance evaluations, we explained the characteristics of the proposed trajectory and confirmed the superiority of the proposed scheme over the conventional schemes in terms of the max–min available energy. We expect that our study will provide a new direction in the design of UAV strategies to increase the capability of EH for PS-based SWIPT systems. An interesting topic for future work is the consideration of a nonlinear energy harvesting model for the UAV-aided SWIPT system.

## Data Availability

Not applicable.
